# Supplementary figures and images for: MPLasso: Inferring microbial association networks using prior microbial knowledge
Source: PLoS Comput Biol. 2017 Dec 27;13(12):e1005915. doi: 10.1371/journal.pcbi.1005915 (PMC5760079; doi:10.1371/journal.pcbi.1005915)

(a)

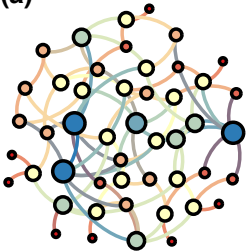

(b)

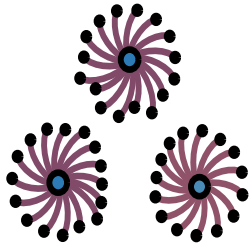

(c)

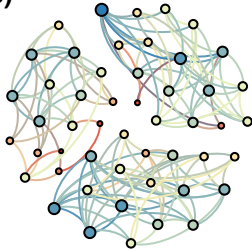

(d)

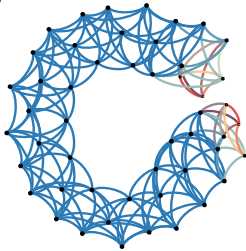

(e)

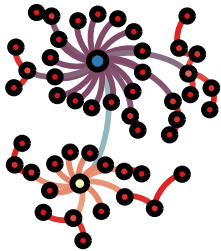

Supplement: S1 Fig — (a) random (b) hub (c) cluster (d) band(4) and (e) scale-free graphs. (PDF) [file pcbi.1005915.s001.pdf]

AUPR

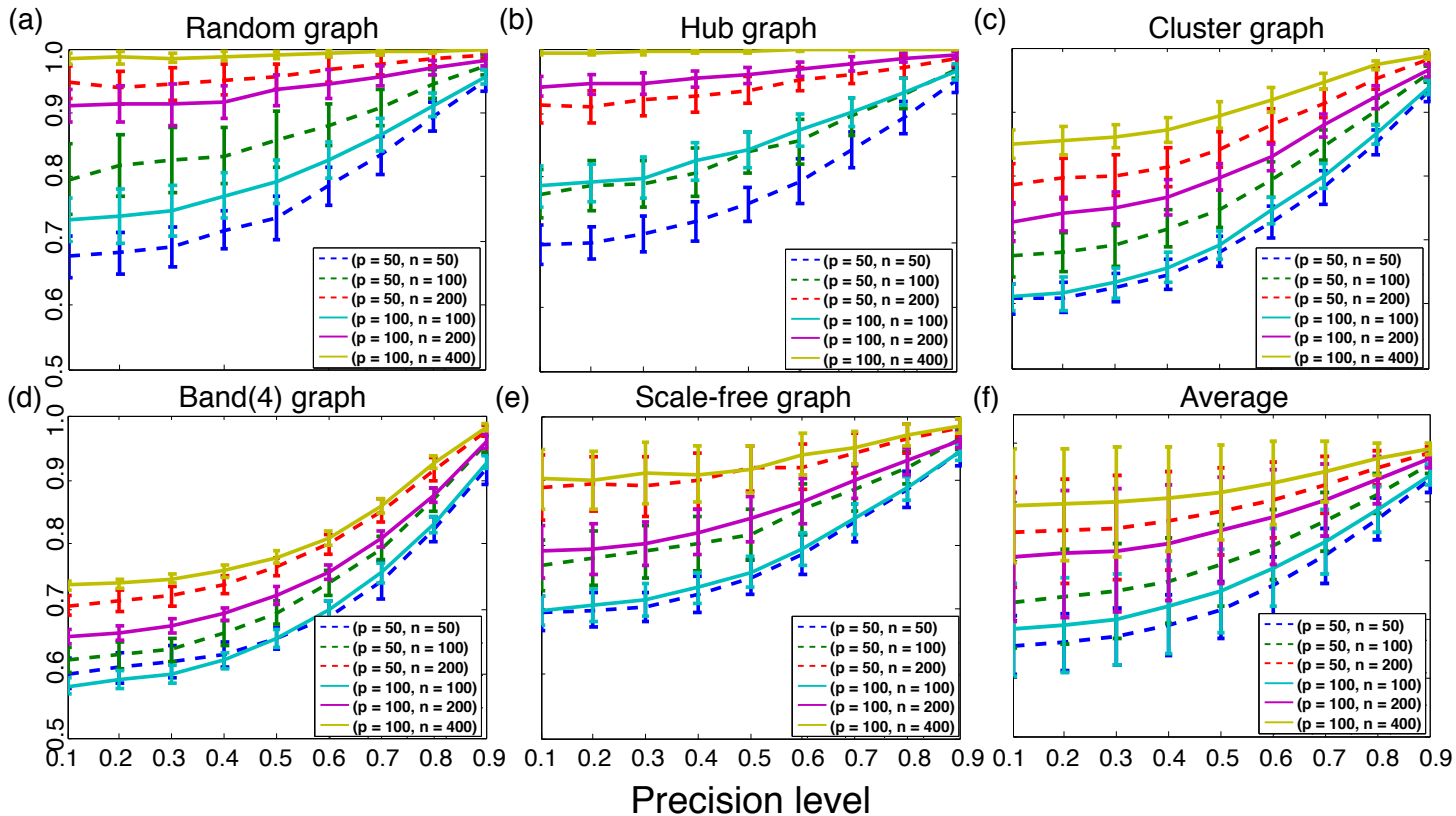

Supplement: S2 Fig — Each point is averaged over 100 simulations. We compare 6 different sets of sample size and OTU numbers ((p = 50, n = (50, 100, 200)) and (p = 100, n = (100, 200, 400)). (PDF) [file pcbi.1005915.s002.pdf]

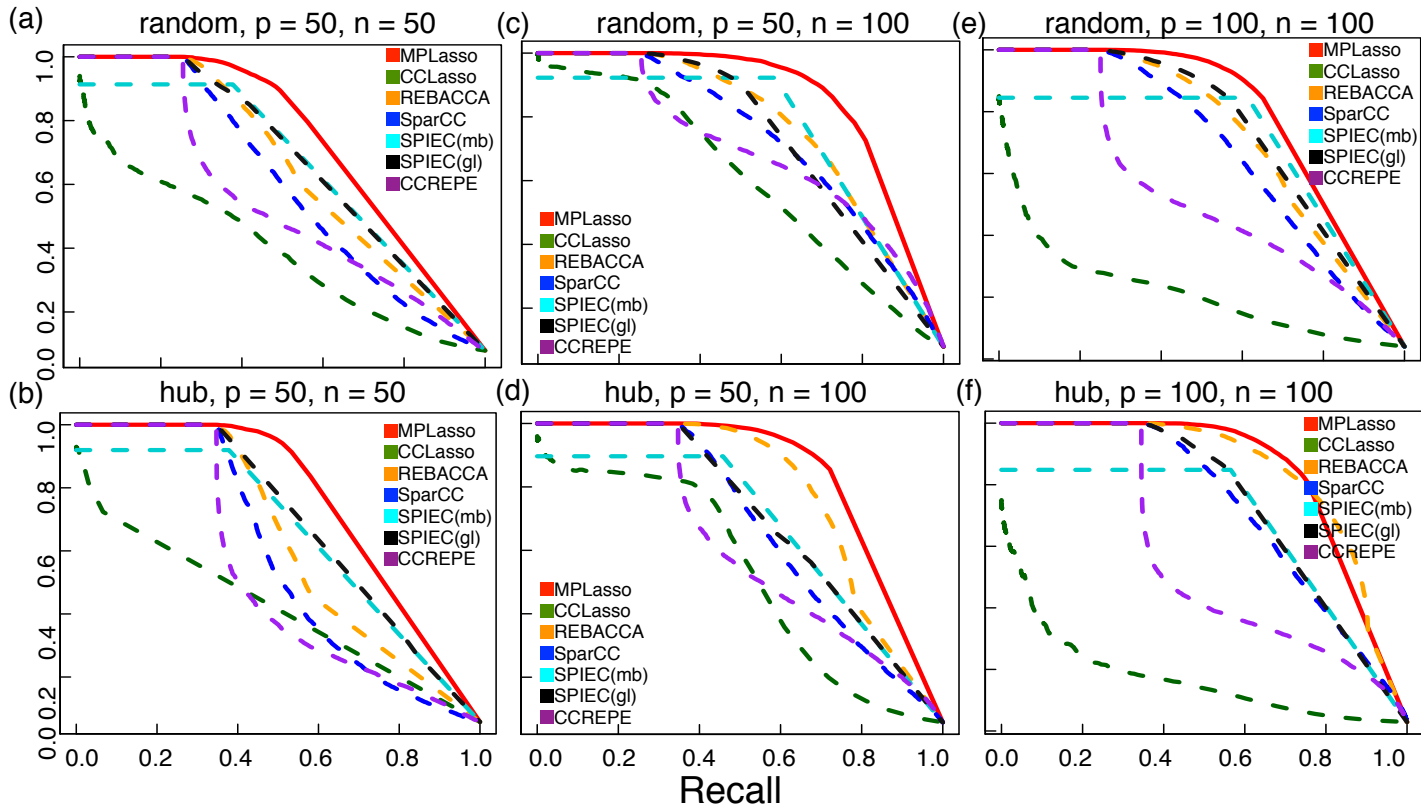

Supplement: S3 Fig — We compare three different sets of sample size and OTU numbers ((p = 50, n = 50), (p = 50, n = 100), and (p = 100, n = 100)). As can be seen the red curve (MPLasso) performs better than all other methods in random and hub graphs. (PDF) [file pcbi.1005915.s003.pdf]

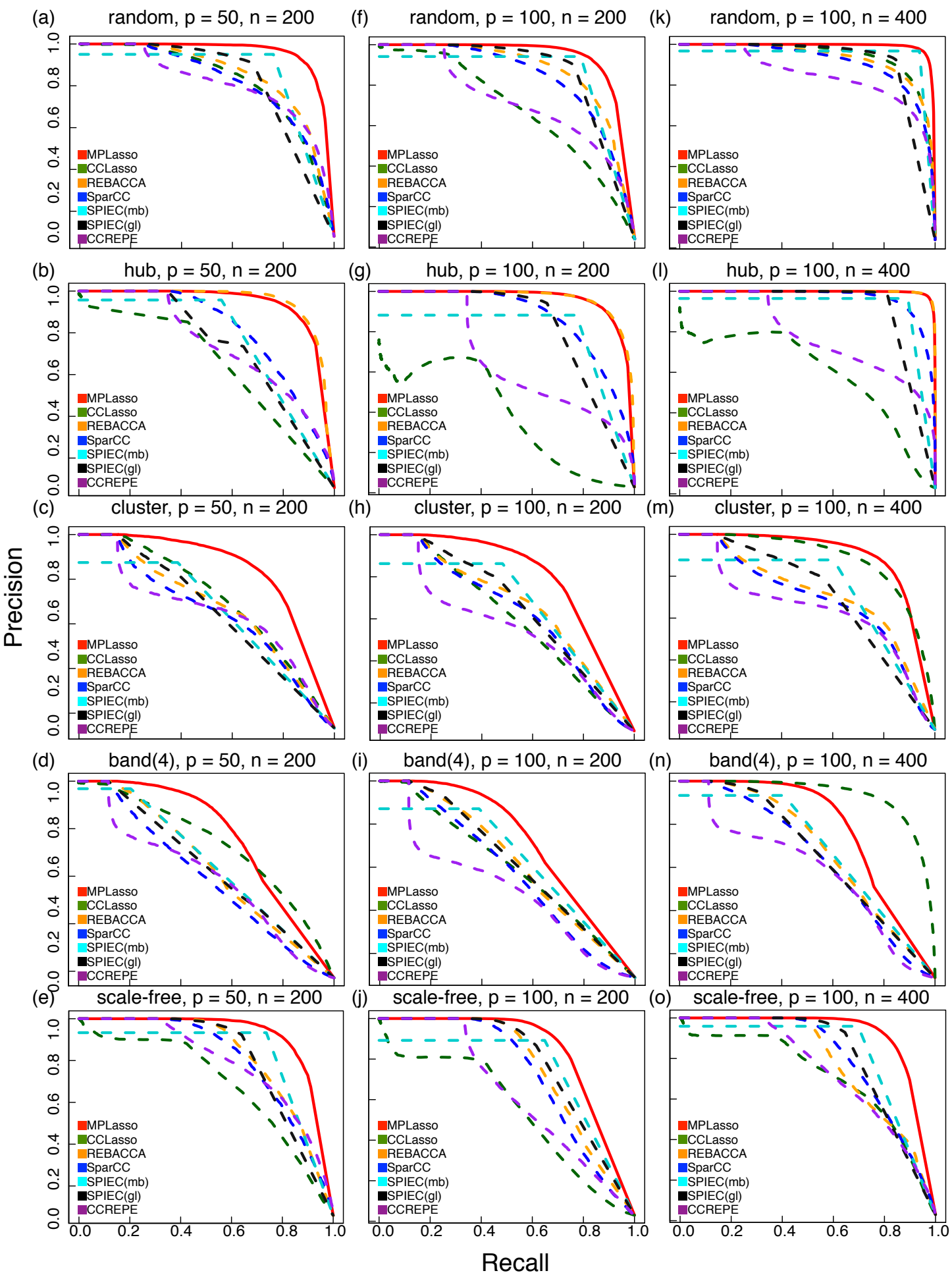

Supplement: S4 Fig — Each set of experiment are averaged over 100 simulations. We compare three different sets of sample size and OTU numbers (i.e., (p = 50, n = 200), (p = 100, n = 200), and (p = 100, n = 400)). As can be seen, the MPLasso (red curve) performs better than all other methods except the band(4) graph when (p = 100, n = 400). (PDF) [file pcbi.1005915.s004.pdf]

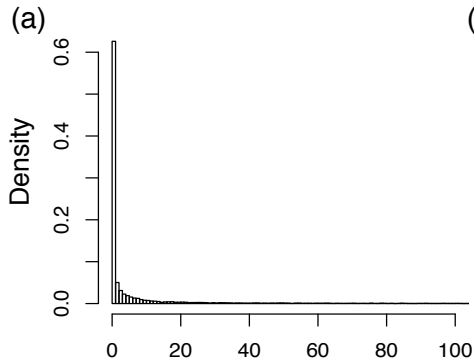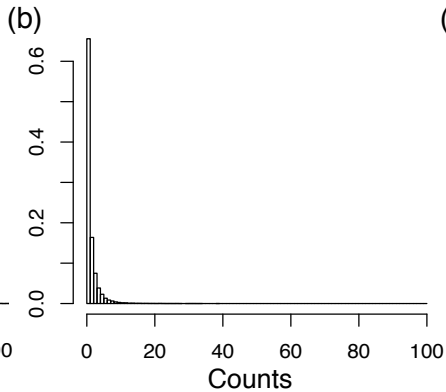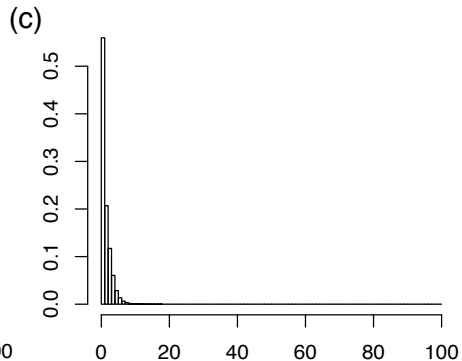

Supplement: S5 Fig — (a) real data (HMMCP, stool samples), (b) additive log-normal distribution, (c) negative binomial distribution. (PDF) [file pcbi.1005915.s005.pdf]

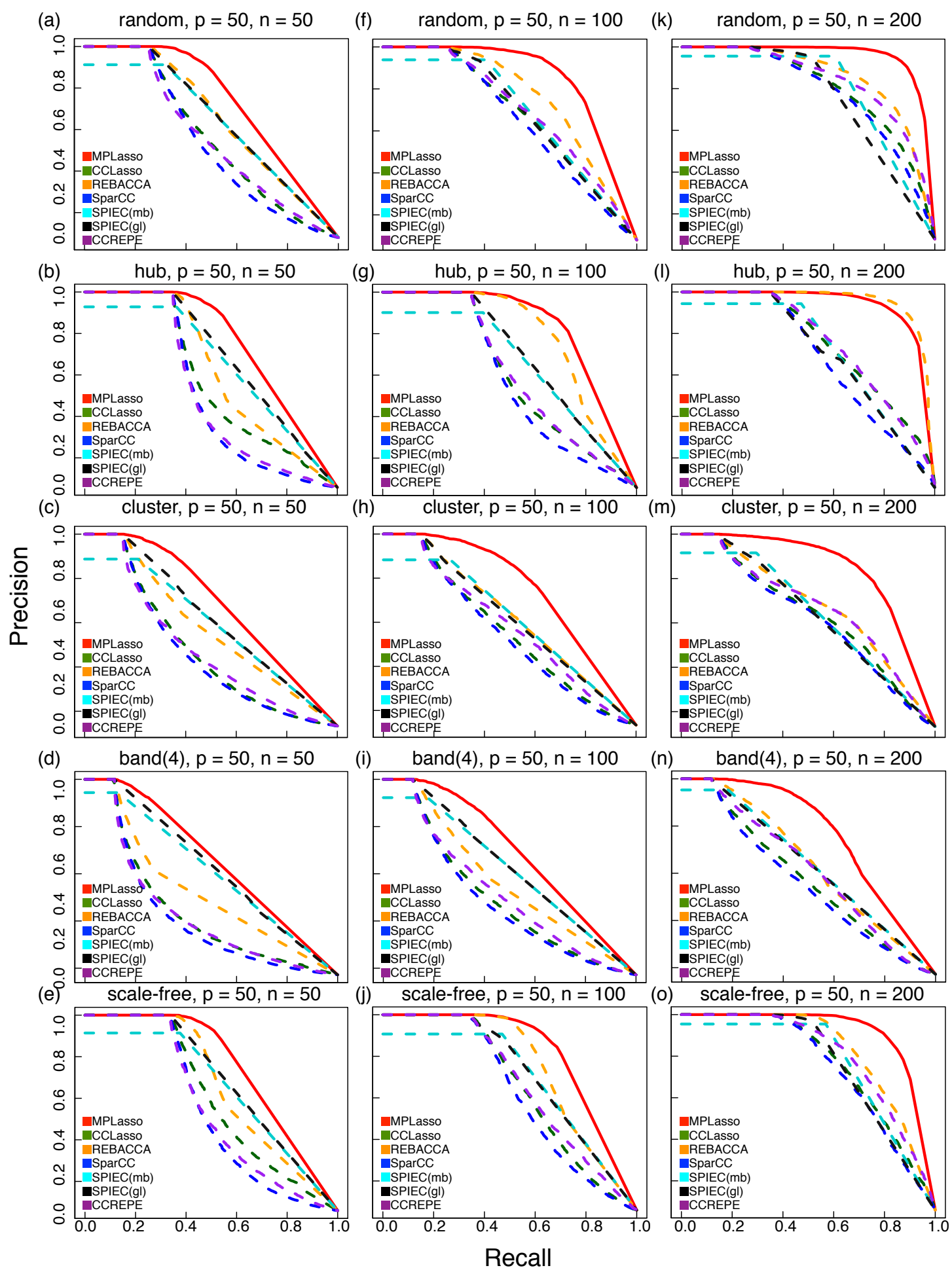

Supplement: S6 Fig — Each set of experiment are averaged over 100 simulations. We compare three different sets of sample size and OTU numbers (i.e., (p = 50, n = 50), (p = 50, n = 100), and (p = 50, n = 200)). As can be seen, the MPLasso (red curve) performs better than all other methods. (PDF) [file pcbi.1005915.s006.pdf]

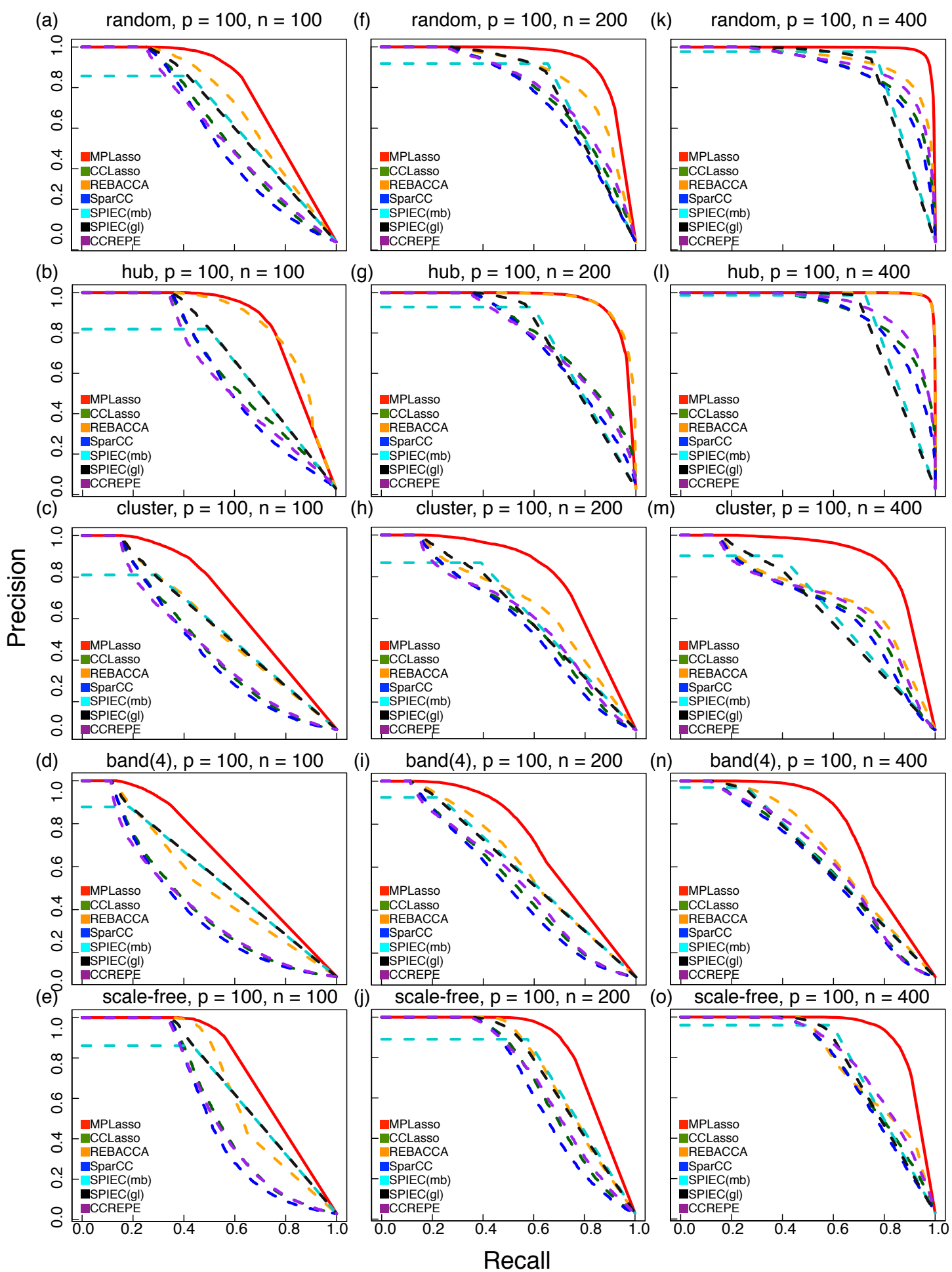

Supplement: S7 Fig — Each set of experiment are averaged over 100 simulations. We compare three different sets of sample size and OTU numbers (i.e., (p = 100, n = 100), (p = 100, n = 200), and (p = 100, n = 400)). As can be seen, the MPLasso (red curve) performs better than all other methods. (PDF) [file pcbi.1005915.s007.pdf]

(a) AntNar, HMASM

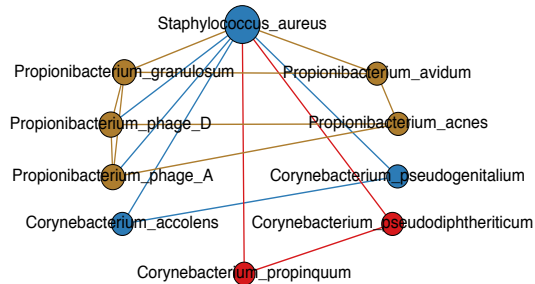

(b) AntNar, HMMCP

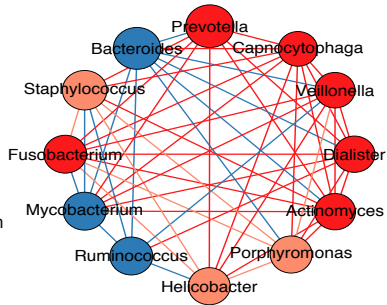

(c) AntNar, HMQCP

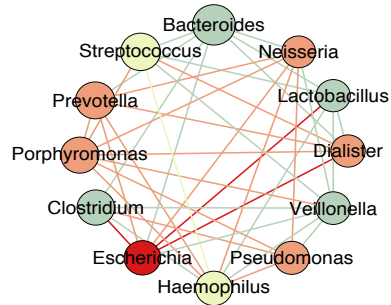

(d) Stool, HMASM

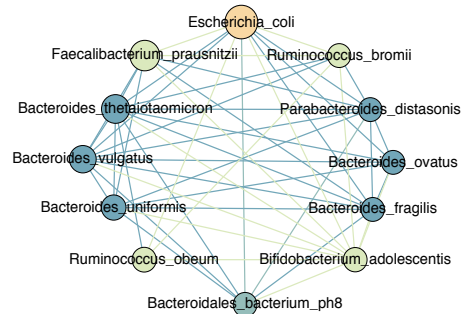

(e) Stool, HMMCP

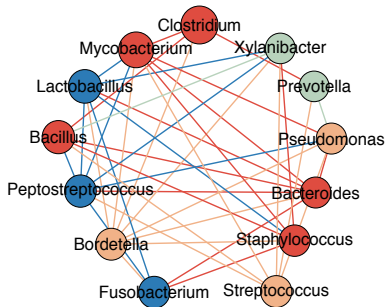

(f) Stool, HMQCP

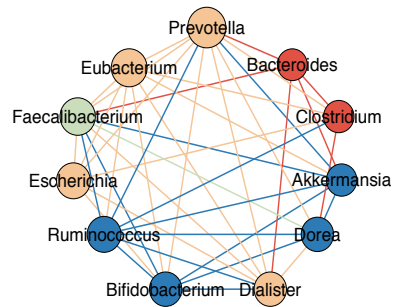

Supplement: S8 Fig — The same node colors represent the communities nodes belong to. As can be seen from species level data (HMASM), phylogenetically related OTUs fall in the same community. Node size represents the relative node degree within the association network with counterclockwise layout. Abbreviations: AntNar: Anterior nares. (PDF) [file pcbi.1005915.s008.pdf]

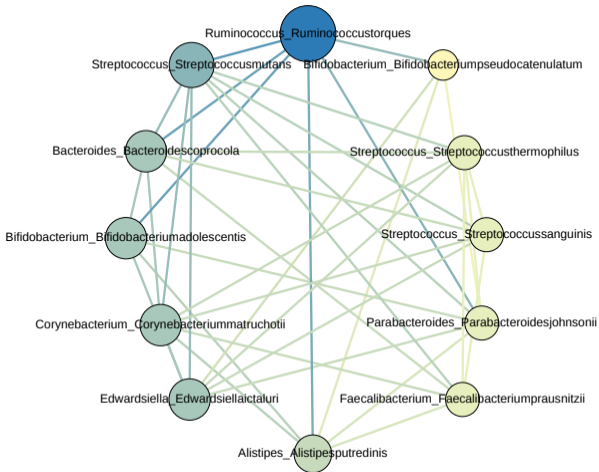

Supplement: S9 Fig — The Global Patterns dataset contains 26 environmental samples and 19216 OTUs. We first use the preprocessing criteria (i.e., OTU variance greater than 10−5) to filter out 32 species. Next, we obtain the prior information which contains 54 interacting taxa pairs from PubMed database. MPLasso finds 155 associated taxa pairs in total. (PDF) [file pcbi.1005915.s009.pdf]

(a)

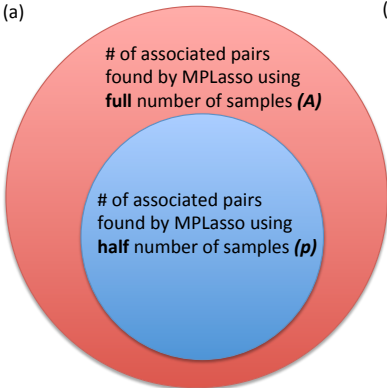

Recovery rate of associated pairs ( $r$ ):  $p/A$

(b)

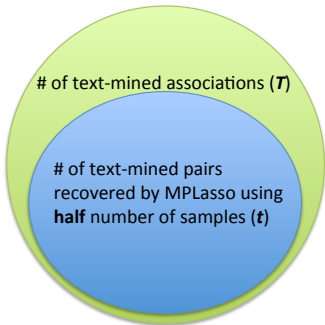

Recovery rate of text-mined pairs ( $s$ ):  $t/T$

Supplement: S10 Fig — (PDF) [file pcbi.1005915.s010.pdf]
