# Supplementary material for: MPLasso: Inferring microbial association networks using prior microbial knowledge
Source: PLoS Comput Biol. 2017 Dec 27;13(12):e1005915. doi: 10.1371/journal.pcbi.1005915 (PMC5760079; doi:10.1371/journal.pcbi.1005915)
Supplement: S1 Table — (PDF) [file pcbi.1005915.s011.pdf]

S1 Table. A comparison of correlation based methods adopted from [1].

Methods	Measures	Assumption	Run-time [secs]
CCREPE	Pearson/Spearman correlation	None	38.284
CCLasso	Pearson correlation of log-ratio transform data	Edge density is no greater than $(\frac{1}{2} - \frac{1}{p-1})$	9.220
SparCC	Pearson correlation of log-ratio transform data	Average correlation of a taxon and others is zero	2.984
REBACCA	Pearson correlation of log-ratio transform data	Each taxon interacts with less than quarter of total taxa	124.840
SPIEC(gl)	Partial correlation of log-ratio transform data	Number of interactions scales linearly with the number of taxa	24.884
SPIEC(mb)	Partial correlation of log-ratio transform data	Number of interactions scales linearly with the number of taxa	20.820
**MPLasso**	Partial correlation of log-ratio transform data	Number of interactions scales linearly with the number of taxa	**1.098**

[1] Li C, et al. Predicting microbial interactions through computational approaches. Elsevier Methods. 2016;102:12?9. doi:10.1016/j.ymeth.2016.02.019.
