# Supplementary material for: MPLasso: Inferring microbial association networks using prior microbial knowledge
Source: PLoS Comput Biol. 2017 Dec 27;13(12):e1005915. doi: 10.1371/journal.pcbi.1005915 (PMC5760079; doi:10.1371/journal.pcbi.1005915)
Supplement: S2 Table — We consider two additional graph structures (random and hub graph) and three sets of parameters, namely, (p = 50, n = 50), (p = 50, n = 100), and (p = 100, n = 100). For each experiment, we average over 100 simulation runs with standard deviations in round brackets. We use three metrics (L1, ACC, AUPR) to quantify the performance. Bold number shows best result. (PDF) [file pcbi.1005915.s012.pdf]

S2 Table. Performance comparison of different methods for additive log normal model.

Method	L_1	ACC	AUPR	L_1	ACC	AUPR	L_1	ACC	AUPR
Random Graph									
MPLasso	**0.069 (0.008)**	**0.956 (0.007)**	**0.735 (0.034)**	0.063 (0.008)	**0.972 (0.007)**	**0.859 (0.044)**	**0.037 (0.004)**	**0.982 (0.003)**	**0.793 (0.034)**
CCLasso	0.080 (0.009)	0.948 (0.008)	0.642 (0.048)	0.068 (0.005)	0.958 (0.008)	0.770 (0.051)	0.051 (0.004)	0.978 (0.003)	0.740 (0.044)
SparCC	0.079 (0.004)	0.946 (0.007)	0.599 (0.044)	0.066 (0.004)	0.954 (0.009)	0.732 (0.049)	0.052 (0.002)	0.975 (0.003)	0.678 (0.043)
REBACCA	**0.069 (0.008)**	0.949 (0.007)	0.652 (0.042)	**0.060 (0.007)**	0.960 (0.009)	0.767 (0.050)	**0.037 (0.004)**	0.978 (0.003)	0.724 (0.044)
SPIEC (mb)	-	0.948 (0.007)	0.653 (0.043)	-	0.962 (0.011)	0.746 (0.046)	-	0.980 (0.003)	0.688 (0.039)
SPIEC (gl)	0.072 (0.008)	0.948 (0.007)	0.679 (0.030)	0.073 (0.009)	0.957 (0.010)	0.746 (0.051)	0.038 (0.004)	0.979 (0.004)	0.745 (0.046)
CCREPE	0.090 (0.007)	0.941 (0.006)	0.546 (0.031)	0.091 (0.007)	0.947 (0.009)	0.694 (0.045)	0.046 (0.003)	0.970 (0.003)	0.540 (0.030)
Hub Graph									
MPLasso	**0.087 (0.001)**	**0.971 (0.003)**	**0.759 (0.027)**	0.086 (0.001)	**0.979 (0.003)**	**0.839 (0.032)**	0.050 (0.001)	**0.989 (0.001)**	**0.842 (0.027)**
CCLasso	0.107 (0.008)	0.964 (0.003)	0.619 (0.049)	0.096 (0.004)	0.972 (0.004)	0.783 (0.047)	0.072 (0.003)	0.986 (0.002)	0.811 (0.037)
SparCC	0.107 (0.002)	0.963 (0.001)	0.573 (0.032)	0.098 (0.001)	0.966 (0.002)	0.674 (0.037)	0.070 (0.001)	0.984 (0.001)	0.725 (0.031)
REBACCA	0.089 (0.002)	0.965 (0.003)	0.633 (0.047)	**0.077 (0.004)**	0.975 (0.005)	0.782 (0.060)	**0.043 (0.002)**	**0.989 (0.002)**	**0.842 (0.042)**
SPIEC (mb)	-	0.961 (0.002)	0.649 (0.067)	-	0.965 (0.003)	0.666 (0.046)	-	0.984 (0.002)	0.669 (0.045)
SPIEC (gl)	0.089 (0.000)	0.963 (0.001)	0.686 (0.018)	0.089 (0.000)	0.965 (0.003)	0.708 (0.027)	0.052 (0.000)	0.985 (0.001)	0.741 (0.023)
CCREPE	0.093 (0.005)	0.962 (0.000)	0.524 (0.023)	0.094 (0.006)	0.962 (0.000)	0.611 (0.029)	0.058 (0.002)	0.981 (0.000)	0.555 (0.025)

We consider two additional graph structures (random and hub graph) and three sets of parameters, namely, $(p = 50, n = 50)$, $(p = 50, n = 100)$, and $(p = 100, n = 100)$. For each experiment, we average over 100 simulation runs with standard deviations in round brackets. We use three metrics (L_1, ACC, AUPR) to quantify the performance. Bold number shows best result.
