# Supplementary material for: MPLasso: Inferring microbial association networks using prior microbial knowledge
Source: PLoS Comput Biol. 2017 Dec 27;13(12):e1005915. doi: 10.1371/journal.pcbi.1005915 (PMC5760079; doi:10.1371/journal.pcbi.1005915)
Supplement: S5 Table — We consider five different graph structures and three sets of parameters, namely, (p = 100, n = 100), (p = 100, n = 200), and (p = 100, n = 400). For each experiment, we average over 100 simulation runs with standard deviations in round brackets. Bold number shows best result. (PDF) [file pcbi.1005915.s015.pdf]

S5 Table. Performance comparison of different methods on negative binomial model.

Method	L_1	ACC	AUPR	L_1	ACC	AUPR	L_1	ACC	AUPR
Random Graph									
MPLasso	**0.036 (0.004)**	**0.982 (0.003)**	**0.783 (0.033)**	0.033 (0.003)	**0.991 (0.003)**	**0.927 (0.028)**	0.028 (0.003)	**0.997 (0.001)**	**0.990 (0.008)**
CCLasso	0.048 (0.005)	0.973 (0.003)	0.609 (0.041)	0.045 (0.002)	0.979 (0.003)	0.777 (0.045)	0.036 (0.002)	0.986 (0.003)	0.893 (0.033)
SparCC	0.054 (0.002)	0.973 (0.003)	0.580 (0.036)	0.045 (0.002)	0.978 (0.003)	0.753 (0.045)	0.038 (0.002)	0.986 (0.003)	0.881 (0.032)
REBACCA	**0.036 (0.004)**	0.977 (0.003)	0.715 (0.038)	**0.032 (0.003)**	0.984 (0.004)	0.855 (0.045)	**0.027 (0.003)**	0.990 (0.003)	0.932 (0.028)
SPIEC (mb)	-	0.974 (0.003)	0.620 (0.044)	-	0.984 (0.003)	0.766 (0.036)	-	0.990 (0.004)	0.866 (0.046)
SPIEC (gl)	0.038 (0.005)	0.974 (0.003)	0.671 (0.023)	0.039 (0.004)	0.983 (0.003)	0.797 (0.041)	0.037 (0.004)	0.989 (0.004)	0.869 (0.050)
CCREPE	0.048 (0.003)	0.972 (0.003)	0.600 (0.041)	0.048 (0.003)	0.980 (0.003)	0.791 (0.042)	0.047 (0.003)	0.988 (0.003)	0.911 (0.029)
Hub Graph									
MPLasso	0.050 (0.001)	**0.990 (0.001)**	0.846 (0.027)	0.047 (0.001)	**0.995 (0.001)**	0.958 (0.015)	0.044 (0.001)	**0.999 (0.001)**	0.996 (0.003)
CCLasso	0.072 (0.003)	0.982 (0.001)	0.648 (0.038)	0.062 (0.002)	0.985 (0.002)	0.796 (0.041)	0.049 (0.001)	0.990 (0.002)	0.904 (0.030)
SparCC	0.074 (0.001)	0.982 (0.000)	0.621 (0.029)	0.064 (0.001)	0.984 (0.001)	0.762 (0.031)	0.057 (0.000)	0.995 (0.001)	0.967 (0.010)
REBACCA	**0.043 (0.001)**	0.989 (0.002)	**0.847 (0.037)**	**0.031 (0.002)**	**0.995 (0.002)**	**0.966 (0.015)**	**0.017 (0.002)**	**0.999 (0.001)**	**0.997 (0.003)**
SPIEC (mb)	-	0.982 (0.001)	0.620 (0.049)	-	0.987 (0.002)	0.746 (0.039)	-	0.968 (0.004)	0.708 (0.035)
SPIEC (gl)	0.052 (0.000)	0.983 (0.001)	0.701 (0.020)	0.052 (0.000)	0.987 (0.001)	0.779 (0.027)	0.089 (0.000)	0.968 (0.004)	0.731 (0.034)
CCREPE	0.060 (0.003)	0.981 (0.000)	0.620 (0.030)	0.062 (0.002)	0.984 (0.001)	0.780 (0.031)	0.103 (0.005)	0.969 (0.003)	0.758 (0.031)
Cluster Graph									
MPLasso	0.029 (0.002)	**0.959 (0.004)**	**0.694 (0.023)**	0.024 (0.002)	**0.970 (0.003)**	**0.802 (0.024)**	0.020 (0.001)	**0.979 (0.003)**	**0.891 (0.023)**
CCLasso	0.051 (0.004)	0.946 (0.003)	0.489 (0.025)	0.044 (0.003)	0.952 (0.003)	0.608 (0.029)	0.036 (0.001)	0.956 (0.004)	0.682 (0.025)
SparCC	0.056 (0.002)	0.946 (0.003)	0.474 (0.024)	0.046 (0.002)	0.951 (0.003)	0.589 (0.028)	0.038 (0.002)	0.955 (0.004)	0.663 (0.026)
REBACCA	**0.025 (0.002)**	0.950 (0.004)	0.582 (0.025)	**0.019 (0.001)**	0.956 (0.004)	0.674 (0.027)	**0.014 (0.001)**	0.959 (0.005)	0.719 (0.026)
SPIEC (mb)	-	0.948 (0.003)	0.546 (0.034)	-	0.955 (0.004)	0.623 (0.026)	-	0.957 (0.004)	0.649 (0.028)
SPIEC (gl)	0.031 (0.002)	0.948 (0.003)	0.584 (0.016)	0.031 (0.003)	0.954 (0.004)	0.641 (0.029)	0.030 (0.002)	0.955 (0.004)	0.650 (0.027)
CCREPE	0.063 (0.005)	0.945 (0.003)	0.482 (0.024)	0.063 (0.005)	0.953 (0.003)	0.620 (0.028)	0.062 (0.005)	0.957 (0.004)	0.695 (0.026)
Band(4) Graph									
MPLasso	0.048 (0.001)	**0.939 (0.003)**	**0.656 (0.015)**	0.041 (0.001)	**0.950 (0.001)**	**0.723 (0.011)**	0.033 (0.001)	**0.959 (0.001)**	**0.779 (0.009)**
CCLasso	0.059 (0.003)	0.925 (0.001)	0.435 (0.014)	0.052 (0.001)	0.932 (0.002)	0.543 (0.013)	0.041 (0.001)	0.939 (0.002)	0.626 (0.010)
SparCC	0.062 (0.001)	0.925 (0.001)	0.419 (0.015)	0.052 (0.001)	0.931 (0.001)	0.523 (0.012)	0.046 (0.001)	0.938 (0.002)	0.611 (0.010)
REBACCA	**0.044 (0.001)**	0.930 (0.002)	0.539 (0.015)	**0.034 (0.001)**	0.939 (0.001)	0.629 (0.011)	**0.027 (0.001)**	0.945 (0.002)	0.684 (0.009)
SPIEC (mb)	-	0.926 (0.001)	0.556 (0.027)	-	0.930 (0.003)	0.602 (0.014)	-	0.934 (0.003)	0.644 (0.016)
SPIEC (gl)	0.050 (0.000)	0.926 (0.001)	0.574 (0.017)	0.050 (0.000)	0.930 (0.002)	0.611 (0.013)	0.050 (0.000)	0.935 (0.003)	0.649 (0.014)
CCREPE	0.091 (0.002)	0.923 (0.001)	0.428 (0.014)	0.093 (0.001)	0.933 (0.002)	0.554 (0.012)	0.095 (0.001)	0.942 (0.002)	0.643 (0.009)
Scale-free Graph									
MPLasso	**0.032 (0.005)**	**0.986 (0.001)**	**0.761 (0.024)**	0.031 (0.004)	**0.990 (0.002)**	**0.846 (0.038)**	0.028 (0.005)	**0.993 (0.002)**	**0.923 (0.036)**
CCLasso	0.042 (0.005)	0.981 (0.001)	0.565 (0.031)	0.041 (0.003)	0.984 (0.001)	0.696 (0.050)	0.036 (0.005)	0.985 (0.001)	0.771 (0.043))
SparCC	0.052 (0.002)	0.981 (0.000)	0.543 (0.028)	0.042 (0.002)	0.983 (0.001)	0.667 (0.045)	0.036 (0.004)	0.985 (0.001)	0.757 (0.044)
REBACCA	0.034 (0.005)	0.984 (0.001)	0.675 (0.027)	**0.030 (0.005)**	0.986 (0.002)	0.756 (0.051)	**0.025 (0.006)**	0.986 (0.002)	0.786 (0.055)
SPIEC (mb)	-	0.981 (0.002)	0.625 (0.060)	-	0.985 (0.002)	0.707 (0.047)	-	0.987 (0.002)	0.772 (0.035)
SPIEC (gl)	0.033 (0.005)	0.982 (0.001)	0.680 (0.025)	0.034 (0.005)	0.985 (0.002)	0.752 (0.034)	0.034 (0.005)	0.987 (0.002)	0.785 (0.034)
CCREPE	0.038 (0.002)	0.981 (0.000)	0.563 (0.034)	0.037 (0.002)	0.983 (0.001)	0.703 (0.049)	0.037 (0.002)	0.986 (0.002)	0.802 (0.043)

We consider five different graph structures and three sets of parameters, namely, ($p = 100$, $n = 100$), ($p = 100$, $n = 200$), and (p=100, n=400). For each experiment, we average over 100 simulation runs with standard deviations in round brackets. Bold number shows best result.
