# Supplementary material for: MPLasso: Inferring microbial association networks using prior microbial knowledge
Source: PLoS Comput Biol. 2017 Dec 27;13(12):e1005915. doi: 10.1371/journal.pcbi.1005915 (PMC5760079; doi:10.1371/journal.pcbi.1005915)
Supplement: S6 Table — Abbreviations: AntNar: Anterior nares, BucMuc: Buccal mucosa, SupPla: Supragingival plague, TonDor: Tongue dorsum. (PDF) [file pcbi.1005915.s016.pdf]

S6 Table. Entry for the 2-by-2 contingency table with the number of abstracts containing neither taxon A nor B in HMP datasets.

	AntNar	BucMuc	Stool	SupPla	TonDor
HMASM	117312	54210	361101	234323	120543
HMMCP	672648	560200	812468	560171	562701
HMQCP	1297841	988084	722368	311344	438151

Abbreviations: AntNar: Anterior nares, BucMuc: Buccal mucosa, SupPla: Supragingival plague, TonDor: Tongue dorsum.
