# Supplementary material for: MPLasso: Inferring microbial association networks using prior microbial knowledge
Source: PLoS Comput Biol. 2017 Dec 27;13(12):e1005915. doi: 10.1371/journal.pcbi.1005915 (PMC5760079; doi:10.1371/journal.pcbi.1005915)
Supplement: S7 Table — This example uses the taxa pair 〈i, j〉 = 〈Escherichia, Citrobacter〉, where ni: the number of abstracts that contains only taxon i, nj: the number of abstracts that contains only taxon j, ni,j: the number of abstracts that contain both taxa i and j, and M: the number of abstracts that contain neither taxa i and j. For this particular contingency table, Fisher’s exact test rejects the hypothesis that taxa i and j are associated. Therefore, these two taxa are not associated with high statistical significance (i.e., the calculated p-value < 0.001) and a higher penalty is placed between taxa i and j in the prior matrix. (PDF) [file pcbi.1005915.s017.pdf]

S7 Table. An illustration example of 2-by-2 contingency table that captures the information about how often two taxa appear together and separately.

$n_{i,j} = 2351$	$n_j = 4750$
$n_i = 349257$	$M = 615268$

This example uses the taxa pair $\langle i, j \rangle = \langle$*Escherichia*, *Citrobacter*$\rangle$, where n_i: the number of abstracts that contains only taxon i, n_j: the number of abstracts that contains only taxon j, $n_{i,j}$: the number of abstracts that contain both taxa i and j, and M: the number of abstracts that contain neither taxa i and j. For this particular contingency table, Fisher's exact test rejects the hypothesis that taxa i and j are associated. Therefore, these two taxa are *not* associated with high statistical significance (i.e., the calculated p-value < 0.001) and a higher penalty is placed between taxa i and j in the prior matrix.
