# Supplementary material for: MPLasso: Inferring microbial association networks using prior microbial knowledge
Source: PLoS Comput Biol. 2017 Dec 27;13(12):e1005915. doi: 10.1371/journal.pcbi.1005915 (PMC5760079; doi:10.1371/journal.pcbi.1005915)
Supplement: S8 Table — For each experiment, we average over 20 simulation runs with standard deviations in round brackets. Bold number shows best result. n and p represent sample size and taxa number, respectively. Abbreviations: AntNar: Anterior nares. (PDF) [file pcbi.1005915.s018.pdf]

S8 Table. Reproducibility for MPLasso, SPIEC (gl), and CCLasso at different body sites of different types of HMP datasets.

Body Site	(n, p)	MPLasso	SPIEC (gl)	CCLasso
HMASM				
AntNar	(91, 14)	**0.917 (0.023)**	0.679 (0.363)	0.902 (0.022)
Stool	(143, 87)	**0.951 (0.005)**	0.912 (0.019)	0.914 (0.006)
HMMCP				
AntNar	(445, 57)	**0.901 (0.006)**	0.734 (0.035)	0.840 (0.013)
Stool	(437, 135)	**0.956 (0.002)**	0.908 (0.004)	0.827 (0.009)
HMQCP				
AntNar	(269, 116)	**0.937 (0.005)**	0.899 (0.005)	0.921 (0.004)
Stool	(319, 64)	**0.894 (0.005)**	0.689 (0.030)	0.806 (0.016)

For each experiment, we average over 20 simulation runs with standard deviations in round brackets. Bold number shows best result. n and p represent sample size and taxa number, respectively. Abbreviations: AntNar: Anterior nares.
