# Supplementary material for: MPLasso: Inferring microbial association networks using prior microbial knowledge
Source: PLoS Comput Biol. 2017 Dec 27;13(12):e1005915. doi: 10.1371/journal.pcbi.1005915 (PMC5760079; doi:10.1371/journal.pcbi.1005915)
Supplement: S9 Table — For each experiment, we average over 20 simulation runs with standard deviations in round brackets. Bold number shows best result. Abbreviations: AntNar: Anterior nares, BucMuc: Buccal mucosa, SupPla: Supragingival plague, TonDor: Tongue dorsum. (PDF) [file pcbi.1005915.s019.pdf]

**S9 Table. Different percentages of top degree nodes to calculate reproducibility for MPLasso, SPIEC (gl) and CCLasso at different body sites of different types of HMP datasets.**

| Body Site | MPLasso | SPIEC (gl) | CCLasso | MPLasso | SPIEC (gl) | CCLasso | MPLasso | SPIEC (gl) | CCLasso |
|---|---|---|---|---|---|---|---|---|---|
| HMASM | top 25% | | | top 50% | | | top 75% | | |
| AntNar | 0.857 (0.057) | 0.633 (0.423) | **0.891 (0.025)** | **0.885 (0.035)** | 0.661 (0.379) | 0.884 (0.021) | 0.897 (0.030) | 0.676 (0.363) | **0.901 (0.021)** |
| BucMuc | **0.929 (0.008)** | 0.847 (0.019) | 0.703 (0.009) | **0.942 (0.006)** | 0.859 (0.020) | 0.730 (0.006) | **0.954 (0.004)** | 0.880 (0.016) | 0.732 (0.005) |
| Stool | **0.894 (0.011)** | 0.820 (0.032) | 0.917 (0.008) | **0.919 (0.008)** | 0.857 (0.026) | 0.913 (0.007) | **0.937 (0.006)** | 0.887 (0.022) | 0.914 (0.007) |
| SupPla | 0.887 (0.008) | 0.794 (0.016) | **0.932 (0.005)** | **0.907 (0.007)** | 0.826 (0.012) | 0.927 (0.005) | **0.925 (0.006)** | 0.852 (0.009) | 0.922 (0.005) |
| TonDor | 0.887 (0.010) | 0.657 (0.029) | **0.928 (0.015)** | **0.916 (0.008)** | 0.692 (0.031) | 0.921 (0.014) | **0.935 (0.006)** | 0.720 (0.031) | 0.918 (0.016) |
| HMMCP | top 25% | | | top 50% | | | top 75% | | |
| AntNar | 0.855 (0.012) | 0.689 (0.044) | **0.851 (0.017)** | **0.867 (0.010)** | 0.706 (0.039) | 0.840 (0.015) | **0.880 (0.008)** | 0.716 (0.037) | 0.835 (0.013) |
| BucMuc | **0.884 (0.010)** | 0.722 (0.037) | 0.851 (0.018) | **0.891 (0.008)** | 0.725 (0.036) | 0.834 (0.015) | **0.905 (0.007)** | 0.740 (0.037) | 0.824 (0.014) |
| Stool | **0.911 (0.006)** | 0.852 (0.007) | 0.858 (0.011) | **0.931 (0.003)** | 0.876 (0.006) | 0.840 (0.009) | **0.944 (0.002)** | 0.892 (0.005) | 0.831 (0.010) |
| SupPla | **0.880 (0.008)** | 0.814 (0.014) | 0.866 (0.014) | **0.891 (0.005)** | 0.829 (0.011) | 0.854 (0.013) | **0.905 (0.005)** | 0.845 (0.009) | 0.845 (0.013) |
| TonDor | **0.890 (0.007)** | 0.786 (0.019) | 0.881 (0.011) | **0.901 (0.004)** | 0.801 (0.017) | 0.872 (0.011) | **0.917 (0.003)** | 0.807 (0.016) | 0.862 (0.012) |
| HMQCP | top 25% | | | top 50% | | | top 75% | | |
| AntNar | 0.873 (0.011) | 0.807 (0.009) | **0.909 (0.006)** | **0.905 (0.008)** | 0.851 (0.007) | 0.910 (0.006) | **0.922 (0.007)** | 0.880 (0.006) | 0.917 (0.004) |
| BucMuc | 0.813 (0.018) | 0.710 (0.033) | **0.860 (0.010)** | **0.825 (0.008)** | 0.733 (0.028) | 0.844 (0.011) | **0.849 (0.007)** | 0.759 (0.026) | 0.829 (0.013) |
| Stool | **0.843 (0.009)** | 0.602 (0.049) | 0.842 (0.025) | **0.858 (0.007)** | 0.622 (0.040) | 0.821 (0.022) | **0.874 (0.006)** | 0.655 (0.035) | 0.804 (0.020) |
| SupPla | 0.844 (0.019) | 0.754 (0.023) | **0.915 (0.007)** | 0.847 (0.017) | 0.765 (0.019) | **0.896 (0.008)** | 0.860 (0.013) | 0.787 (0.016) | **0.895 (0.007)** |
| TonDor | 0.840 (0.015) | 0.716 (0.029) | **0.863 (0.027)** | 0.846 (0.012) | 0.729 (0.023) | **0.860 (0.022)** | **0.848 (0.011)** | 0.726 (0.022) | 0.847 (0.024) |
| hickhline | | | | | | | | | |

For each experiment, we average over 20 simulation runs with standard deviations in round brackets. Bold number shows best result. Abbreviations: AntNar: Anterior nares, BucMuc: Buccal mucosa, SupPla: Supragingival plague, TonDor: Tongue dorsum.
