# Supplementary material for: MPLasso: Inferring microbial association networks using prior microbial knowledge
Source: PLoS Comput Biol. 2017 Dec 27;13(12):e1005915. doi: 10.1371/journal.pcbi.1005915 (PMC5760079; doi:10.1371/journal.pcbi.1005915)
Supplement: S10 Table — (PDF) [file pcbi.1005915.s020.pdf]

S10 Table. Prior Information and the recovery rate of associated pairs found by MPLasso.

	Non-associated Pairs (Prior Information)	Potentially Associated Pairs	Associated Pairs Found by MPLasso	Recovered Associated Pairs (%)
HMASM				
AntNar	51	36	24	21 (88%)
BucMuc	2137	491	209	164 (78%)
Stool	3033	708	341	232 (68%)
SupPla	6291	1965	976	663 (68%)
TonDor	4083	1170	635	514 (80%)
HMMCP				
AntNar	869	727	452	373 (83%)
BucMuc	1692	936	511	427 (84%)
Stool	6012	2084	721	603 (84%)
SupPla	6291	1195	672	529 (78%)
TonDor	2006	920	658	580 (88%)
HMQCP				
AntNar	4213	2457	768	538 (70%)
BucMuc	1504	1271	819	662 (80%)
Stool	1144	872	620	525 (85%)
SupPla	601	674	420	340 (81%)
TonDor	365	625	469	413 (88%)

The first column corresponds to the prior information obtained from the microbial co-occurrence method. The second column is the estimation of the number of potentially associated pairs. The third column is the number of associated pairs found by MPLasso using full dataset. The forth column is the number of recovered associated pairs; we use half of the full dataset to calculate the recovery rate.
