# Supplementary material for: MPLasso: Inferring microbial association networks using prior microbial knowledge
Source: PLoS Comput Biol. 2017 Dec 27;13(12):e1005915. doi: 10.1371/journal.pcbi.1005915 (PMC5760079; doi:10.1371/journal.pcbi.1005915)
Supplement: S11 Table — (PDF) [file pcbi.1005915.s021.pdf]

S11 Table. Recovery rate of associated pairs found by CCLasso and SPIEC (gl).

	Associated Pairs Found by CCLasso	Reproducible Associated Pairs (%)	Associated Pairs Found by SPIEC (gl)	Recovered Associated Pairs (%)
HMASM				
AntNar	26	21 (80%)	65	60 (92%)
BucMuc	984	868 (88%)	239	152 (63%)
Stool	1425	1252 (87%)	165	126 (76%)
SupPla	4496	4161 (92%)	1009	691 (68%)
TonDor	3005	2816 (93%)	1130	721 (63%)
HMMCP				
AntNar	584	464 (79%)	696	530 (76%)
BucMuc	1293	1089 (84%)	1049	751 (71%)
Stool	4047	3119 (77%)	1213	879 (72%)
SupPla	1735	1459 (84%)	916	682 (74%)
TonDor	1735	1494 (86%)	1005	751 (74%)
HMQCP				
AntNar	1733	953 (54%)	991	656 (66%)
BucMuc	1551	1259 (81%)	1116	814 (72%)
Stool	967	769 (79%)	860	635 (73%)
SupPla	752	656 (87%)	589	447 (75%)
TonDor	615	540 (87%)	589	474 (80%)

The first and third column is the number of pairs found by CCLasso and SPIEC (gl) using full dataset. The second and forth column is the recovered associated pairs; we use half of the full dataset to calculate the recovery rate.
