# Supplementary material for: MPLasso: Inferring microbial association networks using prior microbial knowledge
Source: PLoS Comput Biol. 2017 Dec 27;13(12):e1005915. doi: 10.1371/journal.pcbi.1005915 (PMC5760079; doi:10.1371/journal.pcbi.1005915)
Supplement: S12 Table — (PDF) [file pcbi.1005915.s022.pdf]

S12 Table. Interacting taxa pairs found by automated text-mining methods and association pairs suggested by MPLasso.

Taxa Pairs	Body Sites	Type	Reference
⟨*Fusobacterium nucleatum*, *Treponema denticola*⟩	SupPla	NEG	[1]
⟨*Prevotella intermedia*, *Streptococcus mitis*⟩	SupPla	NEG	[2]
⟨*Fusobacterium periodonticum*, *Treponema vincentii*⟩	SupPla	NEG	[1]
⟨*Streptococcus australis*, *Catonella morbi*⟩	SupPla	NA	MPLasso
⟨*Leptotrichia shahii*, *Leptotrichia wadei*⟩	SupPla	NA	MPLasso
⟨*Streptococcus sanguinis*, *Fusobacterium nucleatum*⟩	BucMuc	NEG	[3]
⟨*Actinomyces naeslundii*, *Fusobacterium nucleatum*⟩	BucMuc	NEG	[4]
⟨*Streptococcus infantis*, *Streptococcus salivarius*⟩	BucMuc	NEG	[5]
⟨*Actinomyces graevenitzii*, *Actinomyces naeslundii*⟩	BucMuc	NA	MPLasso
⟨*Rothia aeria*, *Rothia dentocariosa*⟩	BucMuc	NA	MPLasso
⟨*Actinomyces oris*, *Fusobacterium nucleatum*⟩	TonDor	NEG	[6]
⟨*Prevotella nigrescens*, *Prevotella pallens*⟩	TonDor	NEG	[7]
⟨*Fusobacterium periodonticum*, *Treponema socranskii*⟩	TonDor	NEG	[1]
⟨*Neisseria meningitidis*, *Treponema vincentii*⟩	TonDor	NA	MPLasso
⟨*Leptotrichia buccalis*, *Neisseria meningitidis*⟩	TonDor	NA	MPLasso

NEG means negative interactions. NA means Not Applicable since MPLasso is not able to differentiate the interaction type.

References

1. Kolenbrander PE, et al. Intergeneric coaggregation of oral Treponema spp. with Fusobacterium spp. and intrageneric coaggregation among Fusobacterium spp. Infection and Immunity. 1995;63(12):4584–8.

2. Van Hoogmoed CG, et al. Reduction of periodontal pathogens adhesion by antagonistic strains. Oral Microbiology and Immunology. 2008;23(1):43–48. doi:10.1111/j.1399-302X.2007.00388.x.

3. Jiang Q, et al. Interactions between Lactobacillus rhamnosus GG and oral micro-organisms in an in vitro biofilm model. BMC Microbiology. 2016;16(1):149. doi:10.1186/s12866-016-0759-7.

4. Periasamy S, et al. Fusobacterium nucleatum ATCC 10953 requires Actinomyces naeslundii ATCC 43146 for growth on saliva in a three-species community that includes Streptococcus oralis 34. Applied and Environmental Microbiology. 2009;75(10):3250–3257. doi:10.1128/AEM.02901-08.

5. Ortiz-Lucas M, et al. EEffect of probiotic species on irritable bowel syndrome symptoms: A bring up to date meta-analysis. Revista Espanola de Enfermedades Digestivas. 2013;105(1):19–36.

6. Periasamy S, Kolenbrander PE. Central Role of the Early Colonizer Veillonella sp. in Establishing Multispecies Biofilm Communities with Initial, Middle, and Late Colonizers of Enamel. Journal of Bacteriology. 2010;192(12):2965–2972. doi:10.1128/JB.01631-09.

7. Gursoy UK, et al. Prevotella intermedia ATCC 25611 targets host cell lamellipodia in epithelial cell adhesion and invasion. Oral Microbiology and Immunology. 2009;24(4):304–309. doi:10.1111/j.1399-302X.2009.00510.x.
