# Supplementary material for: MPLasso: Inferring microbial association networks using prior microbial knowledge
Source: PLoS Comput Biol. 2017 Dec 27;13(12):e1005915. doi: 10.1371/journal.pcbi.1005915 (PMC5760079; doi:10.1371/journal.pcbi.1005915)
Supplement: S13 Table — (PDF) [file pcbi.1005915.s023.pdf]

S13 Table. Accuracy of edge sign recovery accuracy for different algorithms on different synthetic graph structures.

	MPLasso	CCLasso	REBACCA	SparCC	SPIEC (mb)	SPIEC (gl)	CCREPE
$n = 50, p = 50$							
Random	1.00	0.99	1.00	0.99	0.82	1.00	1.00
Cluster	1.00	0.90	1.00	0.90	0.72	1.00	1.00
Hub	1.00	0.99	1.00	0.99	0.89	1.00	1.00
Scale-free	1.00	0.98	1.00	0.94	0.96	1.00	1.00
Band(4)	1.00	0.90	1.00	0.87	0.82	1.00	1.00
$n = 100, p = 50$							
Random	1.00	1.00	1.00	1.00	0.94	1.00	1.00
Cluster	1.00	0.93	1.00	0.91	0.91	1.00	1.00
Hub	1.00	1.00	1.00	1.00	0.95	1.00	1.00
Scale-free	1.00	0.99	1.00	1.00	0.95	1.00	1.00
Band(4)	1.00	0.90	1.00	0.90	0.83	1.00	1.00
$n = 100, p = 100$							
Random	1.00	1.00	1.00	1.00	0.96	1.00	1.00
Cluster	1.00	0.98	1.00	0.94	0.97	1.00	1.00
Hub	1.00	1.00	1.00	1.00	0.99	1.00	1.00
Scale-free	1.00	1.00	1.00	0.99	0.90	1.00	1.00
Band(4)	1.00	0.90	1.00	0.90	0.83	1.00	1.00
$n = 200, p = 200$							
Random	1.00	1.00	1.00	1.00	1.00	1.00	1.00
Cluster	1.00	1.00	1.00	0.99	1.00	1.00	1.00
Hub	1.00	1.00	1.00	1.00	1.00	1.00	1.00
Scale-free	1.00	1.00	1.00	1.00	0.94	1.00	1.00
Band(4)	1.00	0.95	1.00	0.90	1.00	1.00	1.00

n and p represent number of samples and number of taxa, respectively.
