# Supplementary material for: MPLasso: Inferring microbial association networks using prior microbial knowledge
Source: PLoS Comput Biol. 2017 Dec 27;13(12):e1005915. doi: 10.1371/journal.pcbi.1005915 (PMC5760079; doi:10.1371/journal.pcbi.1005915)
Supplement: S14 Table — (PDF) [file pcbi.1005915.s024.pdf]

S14 Table. Jaccard index of inferred edges among pairwise datasets

	HMMCP-HMQCP	HMMCP-HMASM	HMQCP-HMASM
AntNar	4.0%	1.2%	3.6%
BucMuc	8.0%	10.1%	5.8%
Stool	3.6%	2.4%	0.6%
SupPla	7.8%	20.4%	13.6%
TonDor	16.6%	18.4%	22.8%

Since both HMMCP and HMQCP datasets can only raise up to the genus level, we discard the species names in HMASM dataset when computing the Jaccard index. As can be seen, SupPla and TonDor share around 15-20% of the total edges. However, AntNar and Stool show different results where the fraction of shared edges is less than 10% of the total edges; this may be due to the variations in the number of taxa detected while using different pipelines. For example, for Stool, HMMCP detects 135 taxa, while HMQCP detects only 64 taxa. Other than that, the number of common taxa among HMMCP and HMQCP is only 34; this can greatly reduce the common shared edges among different pipelines.
