# Supplementary material for: MPLasso: Inferring microbial association networks using prior microbial knowledge
Source: PLoS Comput Biol. 2017 Dec 27;13(12):e1005915. doi: 10.1371/journal.pcbi.1005915 (PMC5760079; doi:10.1371/journal.pcbi.1005915)
Supplement: S1 File — Contents: 1. Algorithms summaries, simulation settings and run time comparisons. 2. Graph generation process. 3. The impact of different precision levels on prior matrix and synthetic experiments. 4. Experiments with synthetic data generated from negative binomial distribution. 5. Experiments with HMP datasets with two more body sites. 6. Methods for calculating Spearman correlation of node degrees. 7. Prior knowledge introduction in synthetic experiment. 8. Consistency analysis for graphical Lasso algorithm. 9. Definitions for recovery rate of associated pairs and text-mined pairs. (PDF) [file pcbi.1005915.s025.pdf]

Supplementary Text for MPLasso: Inferring Microbial Association Networks Using Prior Microbial Knowledge

Chieh Lo[1*], Radu Marculescu[1]

1 Department of Electrical and Computer Engineering, Carnegie Mellon University, Pittsburgh, PA, United States of America

* chiehl@andrew.cmu.edu

1 Algorithms summaries, simulation settings and run time comparisons

We first summarize the existing methods on inferring correlations or association on microbial data (synthetic data in S1 Table). More specifically, we consider the following algorithms: CCREPE, CCLasso, SparCC, REBACCA, SPIEC-EASI, and our proposed MPLasso.

For the synthetic and HMP experiments, the settings for each method is as follows: (1) CCLasso: we use the code[1] with the default settings: the number of bootstraps is set to 20. (2) SparCC: we use the implementation from the SPIEC-EASI package with the default settings: the number of iterations for the inner and outer loop is set to 10 and 20, respectively. (3) REBACCA: we use the code[2] with the default settings: the number of bootstraps is set to 40. (4) SPIEC (mb) and SPIEC (gl): we use the code[3] with the default settings: the number of different regularization parameter values is set to 15 and the number of repetitions for StARS is set to 20. (5) CCREPE: we use the code[4] with the default settings: the number of iterations is set to 1000.

The run time is computed by using a synthetic "random" network with 200 samples and 100 OTUs on Intel(R) Core(TM) i5-2400 CPU, 16 GB MEM. We find that MPLasso is much faster than any of the given methods since MPLasso utilize the R package glasso routine which efficiently solve the Lasso problem. SparCC also runs in a much faster fashion since other methods such as CCLasso consists of several optimization procedures from the cross validation.

[1]https://github.com/huayingfang/CCLasso
[2]http://faculty.wcas.northwestern.edu/∽hji403/REBACCA.html
[3]https://github.com/zdk123/SpiecEasi
[4]http://www.bioconductor.org/packages/devel/bioc/html/ccrepe.html

2 Graph generation process

To evaluate the performance of our model to recover different network structures, we consider five different graph structures (see S1 Fig) and p nodes as discussed in [1] with different precision matrices Θ defined as follows:

- Random Graph: Each pair of off-diagonal elements are set to non-zero with probability $\frac{3}{p}$ which results in about $\frac{3}{2}(p-1)$ edges in the graph.
- Hub Graph: Nodes are randomly partitioned into g groups and within each groups, nodes are connected to the center node which result in $p-g$ edges in the graph.
- Cluster Graph: Nodes are randomly partitioned into g groups and within each groups, nodes are connected with probability P which result in $p(\frac{p}{g-1})(\frac{P}{2})$ edges in the graph.
- Band(4) Graph: Each adjacent pair of off-diagonal elements (i.e., node i and j) are connected if $1 \leq |i-j| \leq b$ (b is the bandwidth) which result in $(2p-1-b)\frac{b}{2}$ in the graph. We use $b=4$ to construct the band(4) graph
- Scale-free Graph: We generate the graph by using Barabasi-Albert algorithm [2]. The initial graph has two connected nodes and each new node is connected to only one node in the existing graph with the probability proportional to the degree of the each node in the existing graph. It results in p edges in the graph.

3 The impact of different precision levels on prior matrix and synthetic experiments

Given different precision levels, the ratios between maximum (M) and minimum (m) values in prior matrix (P) are adjusted. The minimum and maximum value used in the prior matrix is based on the BIC model selection criteria (described in the **main manuscript**). More specifically, the maximum value M is set to $m/(1-p)$ where m is the minimum value selected from BIC and p is the precision level.

We examine how different precision level can affect the performance in terms of AUPR on different graph structures. As shown in S2 Fig, we notice that the performance of MPLasso increases as the precision level increases; this confirms that accurate microbial prior information can help the graph estimation algorithm (i.e., it becomes more accurate on inferring the graph structures). MPLasso cannot estimate well on band(4) graph due to the special structure. However, it can still achieve up to 0.6 AUPR when precision level is 0.1. For real data experiments, we set up a precision level equal to 0.5 and use BIC to choose m and then calculate M to form the prior matrix.

4 Experiments with synthetic data generated from negative binomial distribution

The metagenomics data is often highly zero-inflated and can have large counts. To show that our log-normal distribution assumption for count data is valid, we look into the density distribution of 16S rRNA experimental data and the simulated data of both log-normal and negative binomial distributions. As shown in S5 Fig(a), we first examine the density distribution of the stool samples from the HMMCP dataset; it is obvious that the real data is highly zero-inflated and has a very low density at larger counts (a similar distribution can be found at different body sites).

As shown in S5 Fig(b), the density for the log-normal distribution is also a zero-inflated distribution, similar to the real data density distribution. We also consider another zero-inflated distribution, namely, the negative binomial distribution with the density distribution shown in S5 Fig(c); the results are similar but with a higher density at the locations of smaller counts.

For completeness, we include a new set of experiments to show that our proposed algorithm is able to deal with zero-inflated distributions. More precisely, we choose the negative binomial distribution that is also suitable to model the microbial count data [3].

We consider the same experimental setting of parameters (i.e., different number of taxa, sample sizes, and graph structures) in the main manuscript. As shown in S6-7 Figs and S4-5 Tables, the performance of our proposed method outperforms all the other methods except a few cases involving hub graphs; this is similar to the results for the additive log-normal model. In summary, our results show that MPLasso works well with many different distributions and graph structures.

5 Experiments with HMP datasets with two more body sites

We report two additional body sites in S8 Table and S8 Fig. The reproducibility results shows that MPLasso has a better reproducibility over SPIEC (gl) and CCLasso which is the same as in the main manuscript.

For the anterior nares (AntNar), the HMASM dataset (S8 Fig(a)) only contains 14 taxa since the trimmed sequences are too short for metaphlan2 to extract effective amounts of taxa. On the other hand, HMMCP and HMQCP (S8 Fig(b) and (c)) detect similar genera, for example, *Prevotella*, *Bacteroides*, and *Porphyromonas* which are common in AntNar. The association pairs found in both HMMCP and HMQCP, for example, ⟨*Prevotella*, *Bacteroides*⟩ has been found to have inverse correlation [4].

For the stool HMASM samples (S8 Fig(d)), MPLasso suggests an association between the ⟨*Faecalibacterium prausnitzii*, *Escherichia coli*⟩ pair. Although not yet been validated in laboratory settings, researchers have observed the co-abundance of these two species in the human gut [5]. For the genus level data shown in S8 Fig(e) and (f), only two genera (*Bacteroides* and *Prevotella*) have been found in common in HMMCP and HMQCP datasets; this may be due to the variations of samples and the number of taxa detected using different pipelines (HMMCP detects 135 taxa while HMQCP obtains 64).

We also consider reproducibility on different percentages of highly connected nodes in S9 Table. Only when we consider as little as only 25% of high degree nodes, CCLasso has a better performance (but even so for 2% only, on average). While MPLasso outperforms SPIEC (gl) in all the cases reported in S9 Table.

6 Methods for calculating Spearman correlation of node degrees

The correlation between node degrees at different body sites is calculated by utilizing the Spearman correlation method. More specifically, we first rank a node (i.e., microbe) based on its node degree. Next, we compute the Spearman correlation based on the rank list. For example, for Stool, we first obtain rank list r_1 and r_2 for HMMCP and HMQCP pipelines, respectively. Next, we compute the Spearman correlation among r_1 and r_2. The purpose of calculating the correlation is to compare and show the differences between the two pipelines and how consistent our proposed algorithms is when inferring the node degrees.

7 Prior knowledge introduction in synthetic experiment

The procedure of introducing prior knowledge into the algorithm is as follows: For synthetic data, the prior information is obtained by sampling the true network structure (i.e., the adjacency matrix) and adding noise to simulate realistic conditions.

First, we choose a percentage (based on the prior percentage parameter) of random edges from the true network structure to be used as prior information (i.e., the percentage of "perfect" prior information being used). For example, if the total number of true edges is 100, then a prior percentage = 50% will randomly choose 50 true edges as our prior information.

Second, in order to account for imprecise information (e.g., wrongly annotated associations in the scientific literature) that may appear in the real datasets, we consider the precision of the prior information. If the precision level parameter is set at 50%, then we randomly replace 50% of the correct prior information (25 true edges following our example) with false edges to simulate imprecise information.

8 Consistency analysis for graphical Lasso algorithm

Let $X \in \mathbb{R}^{n \times p}$ be the compositional data after applying the *clr* transformation, where n represents the total number of samples. Let C^* be the true (i.e., not observed) covariance matrix of the compositional data and $\hat{C} = \text{Cov}[X]$ be the empirical covariance matrix of the compositional data. It has been shown in [6] that if $||\hat{C} - C^*||_{\max} \preceq \sqrt{\frac{\log p}{n}}$ and the penalizing parameter (λ) is selected as $\lambda \succeq \sqrt{\frac{\log p}{n}}$, then $||\hat{\Omega} - \Omega^*||_{\max} \preceq (\sqrt{\frac{\log p}{n}} + \lambda)$, where $\hat{\Omega}$ is the estimated precision matrix (i.e., inferred associations) and Ω^* is the true precision matrix (i.e., underlying true associations)[5].

For example, if $n = 200, p = 200$, we get $\sqrt{\frac{\log p}{n}} = 0.16$. If we choose $\lambda > 0.16$, we get the l_∞ error for the precision matrix which will be less than 0.32. This guarantees that we can have a consistency estimator on the precision matrix. We can also observe that the approximation error ($||\hat{C} - \Gamma||_{\max} < \frac{1}{p} = 0.005$) is relatively small compared to the estimation error for the precision matrix (Ω). Hence, the approximate error of compositional data has little effect on the inference results.

[5] $||.||_{\max}$ denotes the element-wise l_∞ norm.

9 Definitions for recovery rate of associated pairs and text-mined pairs

The S10 Table reports the statistics of the recovery rate of “associated” pairs; this is different from the recovery rate of the “text-mined” interactions. More specifically, as shown in S11 Fig.(a), the recovery rate (r) of associated pairs is defined as: $r = \frac{p}{A}$, where p represents the number of associated pairs found by MPLasso using *half* of the samples in full dataset, while A represents the number of the associated pairs found by MPLasso using the *full* dataset.

On the contrary, as shown in S11 Fig.(b), the recovery rate of the text-mined associations (s) is defined as: $s = \frac{t}{T}$, where t represents the number of text-mined associations recovered by MPLasso, and T represents the number of text-mined associations found using text-mining methods.

References

1. Zhao T, et al. The huge package for high-dimensional undirected graph estimation in R. Journal of Machine Learning Research. 2012;13:1059–1062.

2. Albert R, Barabási A. Statistical mechanics of complex networks. Reviews of modern physics. 2002;74(January).

3. McMurdie PJ, Holmes S. Waste Not, Want Not: Why Rarefying Microbiome Data Is Inadmissible. PLOS Computational Biology. 2014;10(4):1–12. doi:10.1371/journal.pcbi.1003531.

4. Gorvitovskaia A, et al. Interpreting Prevotella and Bacteroides as biomarkers of diet and lifestyle. Microbiome. 2016;4:15. doi:10.1186/s40168-016-0160-7.

5. Lopez-Siles M, et al. Mucosa-associated Faecalibacterium prausnitzii and Escherichia coli co-abundance can distinguish Irritable Bowel Syndrome and Inflammatory Bowel Disease phenotypes. International journal of medical microbiology. 2014;304(3-4):464–75. doi:10.1016/j.ijmm.2014.02.009.

6. Loh P, Wainwright MJ. Structure Estimation for Discrete Graphical Models: Generalized Covariance Matrices and Their Inverses. The Annals of Statistics. 2013;41(6):3022–3049.
